# Isolated cerebral mucormycosis masquerading as a tumor in an immunocompetent patient

**DOI:** 10.4322/acr.2020.233

**Published:** 2021-01-20

**Authors:** Smita Chandra, Shubhi Sharma, Ruchir Vats, Sanjeev Pandey

**Affiliations:** 1 Swami Rama Himalayan University, Himalayan Institute of Medical Sciences, Department of Pathology, Dehradun, Uttarakhand, India; 2 Swami Rama Himalayan University, Himalayan Institute of Medical Sciences, Department of Neurosurgery, Dehradun, Uttarakhand, India

**Keywords:** Cerebral Cortex, Immunocompromised Host, Mucormycosis

## Abstract

Mucormycosis is an opportunistic fungal disease that commonly presents as cutaneous or rhinocerebral infections associated with immunocompromised states. It may exceptionally present as isolated involvement of the brain with a varied clinical presentation, which may be difficult to diagnose early, leading to increased mortality. Herein, we report the case of a 42-year-old immunocompetent female with left-sided limb weakness and a history of recurrent vomiting and headache for the last two years. Clinically, glioma was suspected, but histopathological examination revealed a few broad aseptate fungal hyphae. As no other organ was involved, the diagnosis of isolated cerebral mucormycosis was rendered. Reporting this case, we show an unusual presentation of a central nervous system mucormycosis masquerading a tumor in an immunocompetent patient. The case also highlights the importance of a careful histopathological examination to avoid missing the presence of occasional fungal hyphae. Ideally, recognition of fungal hyphae in the brain, during intraoperative consultation, can prompt brain tissue culture for definitive diagnosis and early empirical antifungal therapy, which may prove life-saving.

## INTRODUCTION

Mucormycosis is an opportunistic infection caused by a filamentous fungus of Mucorales order with rhizopus, mucor, and lichtheimia species being the most common reported pathogens.^1^ It commonly presents as cutaneous, sinuses, or pulmonary infections with the possible mode of entry due to inhalation of spores, inoculation of skin, or ingestion of contaminated food.^2^ ^,^ ^3^ It is usually associated with immunocompromised states like diabetes mellitus, malignancies, or organ transplantation with a high mortality rate.^3^ Cerebral mucormycosis mostly occurs due to the extension of the disease from the nasal cavity, sinuses, and orbit and is referred to as rhinocerebral mucormycosis accounting for 30% of cases.^4^ However, very rarely, it may present as isolated involvement of the brain without any evidence of extension from neighboring structures.^4^ ^,^ ^5^ This exceptional isolated cerebral presentation may have a varied clinical presentation, which may be difficult to diagnose early, leading to increased mortality.

The present case of mucormycosis is, therefore, being reported because the rare presentation of this infection, mimicking tumor, can lead to delayed diagnosis and death of the patient.

## CASE REPORT

**Figure 1:**
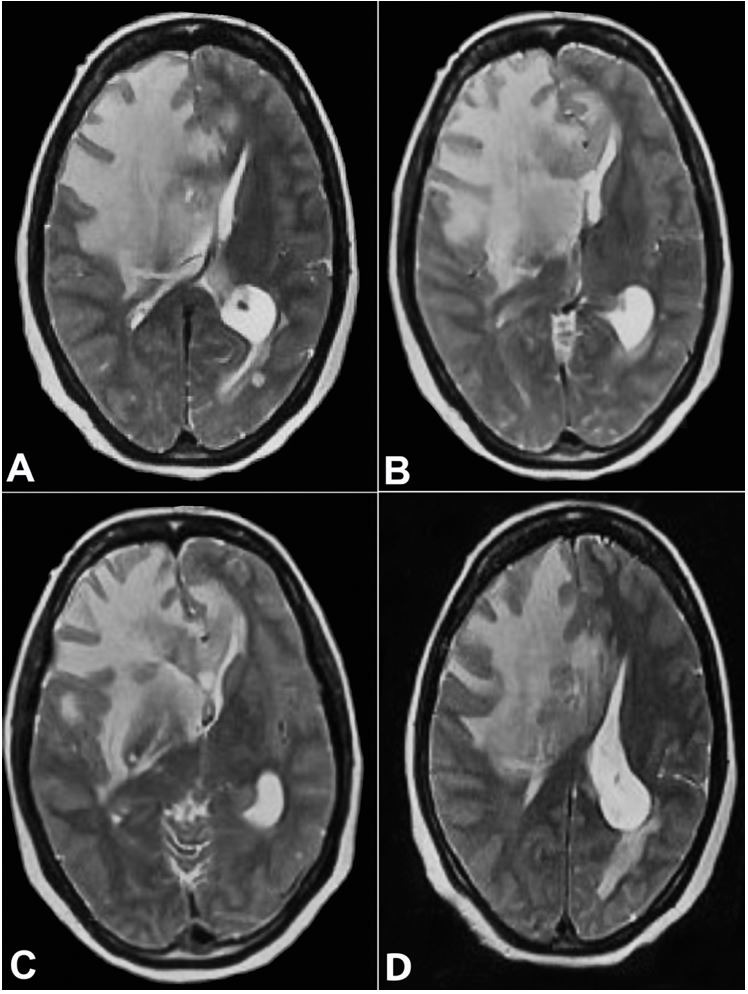
MRI axial T2 W image at the level of basal ganglia and body of lateral ventricle showing diffuse edema at the right frontal white matter and capsule-ganglionic region with expansion of frontal lobe, compression of ipsilateral lateral ventricle and contralateral midline shift along with focal areas intermediate signal intensity noted at the right peri-frontal region and corpus callosum.

**Figure 2:**
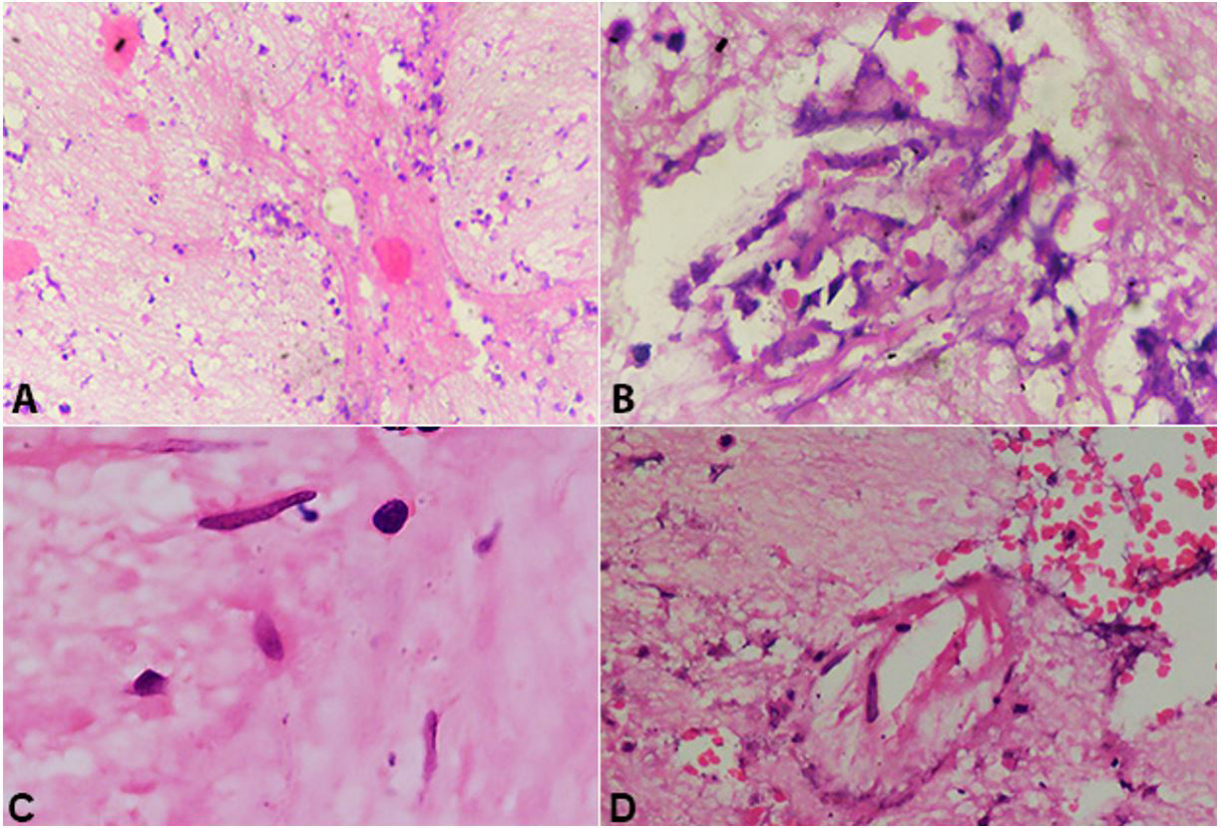
Photomicrographs of the brain tissue. **A** – Gliosis with inflammatory cells and focal necrosis (H&E, x10); **B** and **C** – Broad, aseptate, obtuse angle branching hyphae in necrotic background (H&E, x20, x40 respectively); **D** – broad aseptate branching hyphae showing angio-invasion (H&E, x40).

A 42-year-old female presented with complaints of left-sided limb weakness, multiple episodes of headache, and vomiting over the last 6 months, accompanied by a low-grade fever over the last two days. There was no history of any head trauma, hypertension, diabetes mellitus, tuberculosis, blood transfusion, steroid usage, other chronic illnesses, drug abuse, or any previous surgery. On clinical examination, the patient was conscious, oriented, and was following verbal commands. Higher cerebral functions, cranial nerves were intact, and muscular strength was normal in all limbs. Her hematological findings revealed hemoglobin of 10.66 g/dl (reference range of 12 to 15 g/dl), packed cell volume (hematocrit) 33.06%, total leucocyte count of 5,500/mm^3^ (reference range; 4,000 to 10,000/mm^3^), differential leucocyte count of 71% neutrophils, 23% lymphocytes, 4% monocytes and 2% eosinophils, and erythrocyte sedimentation rate of 46mm/hour. All the biochemical findings, including liver and kidney function tests and serum electrolytes, were within the normal range. Chest X-Ray and abdominal ultrasonography were normal. The brain Magnetic resonance imaging (MRI) showed a space-occupying mass in the right frontal lobe, involving the caudate nucleus, genu, and body of corpus callosum (Figure 1). Sinuses and orbits were free of disease. Based on clinical and radiological findings, the diagnosis of glioma was considered. Right frontal craniotomy was performed. The intraoperative findings included a partly soft and partly firm mass, partly liquefied and suctionable, poorly vascularized with no necrosis. Histopathological examination revealed gliosis moderate lymphocytic and neutrophilic infiltration and perivascular infiltration of plasma cells (Figure 2A). The glial tissue did not show increased cellularity, pleomorphism, atypical mitosis or endothelial proliferation for diagnosis of astrocytoma. However, on careful examination of the H&E stained slides, a few fungal hyphae were seen, which were broad and aseptate, with obtuse angle branching along with focal necrosis and angioinvasion (Figure 222D). Gomori methenamine silver (GMS) stain confirmed the presence of mucormycosis with spores (Figure 33B), and the diagnosis of cerebral mucormycosis was rendered. As there was no evidence of any other organ involvement, so it was considered a case of isolated cerebral involvement. Immediate intravenous 5mg/kg/day of liposomal Amphotericin B was started, and the patient responded well to the treatment and is presently asymptomatic for the last six months.

**Figure 3:**
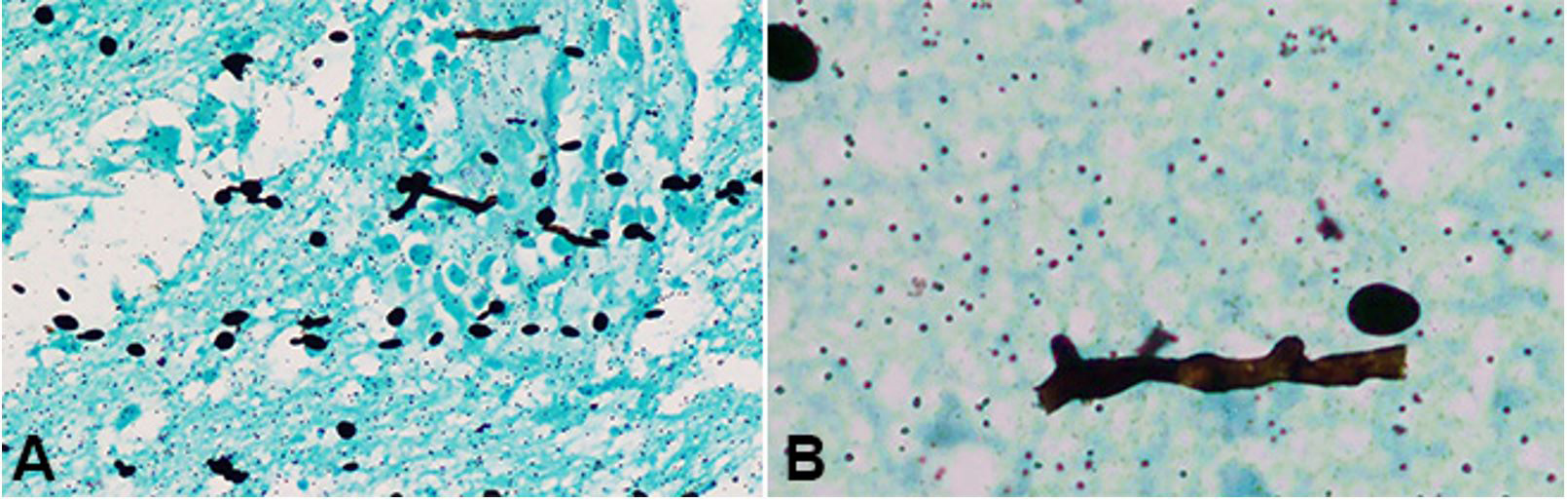
Photomicrographs of the brain tissue. **A** and **B** – Broad, aseptate, obtuse angle branching hyphae with spores in glial tissue (Gomori methenamine silver stain, x20, x100 respectively).

## DISCUSSION

Isolated cerebral mucormycosis, a sporadic presentation, accounts for 8% of total cases of mucormycosis.^5^ The possible mode of infection is the inhalation of spores, which reaches the cerebral tissue without involving other organs by invading the bloodstream. The species of the Mucorales order attaches to the endothelium by specific receptors, followed by endocytosis and angioinvasion.^6^ The basal ganglia are involved in 72.3% of isolated cerebral mucormycosis as the fungus spreads by hematogenous seeding through the middle cerebral artery perforating branches,^7^ as observed in the present case.

Typical cerebral mucormycosis occurs due to the extension of the paranasal sinuses infection associated with immunosuppression, and 82% of the cases of isolated cerebral mucormycosis occurs due to intravenous drug abuse.^7^ This increased risk highlights the importance of a thorough clinical history and examination for intravenous drug abuse in all the isolated cerebral mucormycosis cases. Hematological malignancies have also been reported to be rarely associated with isolated cerebral mucormycosis.^8^ However, the present case exceptionally failed to show any evidence of intravenous drug abuse or other known cause of immunosuppression, pointing out the importance of clinical suspicion of isolated cerebral mucormycosis in immunocompetent cases. The literature search shows that rarely isolated cerebral mucormycosis may be seen in immunocompetent patients. Gupta et al.^9^ reviewed six cases of isolated cerebral mucormycosis, including two pediatric cases. Al Barbarawi et al.^10^ reported a case of isolated cerebral mucormycosis in an immunocompetent 4-year-old child.

Another important feature of our case was the initial suspicion of cerebral tumors based on the clinical presentation and the imaging examination. Dussaule et al.^11^ observed mass effect and edema with peripheral serpiginous and radial strands enhancements on brain MRI in isolated cerebral mucormycosis. Occasional mucormycosis cases have been reported in the literature that masquerades a tumor and thus raises awareness of the varied presentation of this infection.^12^ ^,^ ^13^ Yang et al.^14^ reported a case of pulmonary mucormycosis in a 45-year-old immunocompetent man mimicking lung carcinoma. Similarly, Malek et al.^15^ also reported a case of fatal disseminated mucormycosis mimicking malignancy.

The definite diagnosis of isolated cerebral mucormycosis may pose a problem as culture may be negative in 62% of cases.^7^ However, the diagnosis may be made using a molecular technique like next-generation sequencing (NGS) or polymerase chain reaction (PCR) to detect the fungus in cerebrospinal fluid.^5^ ^,^ ^16^ ^,^ ^17^ Unfortunately, due to financial constraints, molecular techniques may not be utilized in resource-limited settings, and histopathological examination remains the gold standard for diagnosis of isolated cerebral mucormycosis. The present case was also diagnosed on histopathological examination by carefully examining the H&E stained and GMS stained slides. The histomorphological presence of gliosis with neutrophils, plasma cells, vasculitis, and necrosis may give a clue for this fungal infection, which may be confirmed by demonstrating broad, aseptate, and obtuse-angled branched fungal hyphae. Another important feature that may vigilantly be searched in the sections is the presence of angioinvasion, as mucormycosis has a predilection for the endothelium. At times, the diagnosis is made on autopsy.^18^

The mortality rate of the isolated cerebral mucormycosis cases is high and rapid and is reported to reach 65% and within 20 days of admission to the hospital.^4^ ^,^ ^5^ ^,^ ^7^ However, the present case responded well to amphotericin B treatment, and the outcome was favorable, probably by the absence of a risk factor or immunosuppression and the timely diagnosis.

## CONCLUSION

The case highlights the unusual presentation of a rare case of isolated cerebral mucormycosis mimicking a CNS tumor in an immunocompetent patient with the absence of any risk factors and chronic history. Careful histopathological examination for the identification of fungal hyphae and associated subtle features is helpful in its diagnosis. The definite early diagnosis followed by prompt treatment proves beneficial to prevent mortality in this otherwise fatal disease.
